# Diagnostic tests for ovarian cancer in premenopausal women with non-specific symptoms (ROCkeTS): prospective, multicentre, cohort study

**DOI:** 10.1136/bmj-2024-083912

**Published:** 2026-01-29

**Authors:** Sudha Sundar, Ridhi Agarwal, Katie Scandrett, Clare Davenport, Ben Van Calster, Susanne Johnson, Partha Sengupta, Radhika Selvi-Vikram, Fong Lien Kwong, Sue Mallett, Caroline Rick, Sean Kehoe, Dirk Timmerman, Tom Bourne, Hilary Stobart, Richard D Neal, Usha Menon, Aleksandra Gentry-Maharaj, Lauren Sturdy, Ryan Ottridge, Jonathan J Deeks, ROCkeTS collaborators, Ahmed Abdelbar, Shahram Abdi, Parveen Abedin, Hafez Alawad, Victoria Ames, Moji Balogun, Sarah Baron, Tracey Butcher, Sarah Coleridge, Tim Duncan, Kendra Exley, Ketankumar Gajjar, Fateh Ghazal, Chellappan Gnanachandran, Marianne Hamer, Neil Hebblethwaite, Tracey Hughes, Karen Jermy, Susanne Johnson, Sonali Kaushik, Patrick Keating, Robert Kent, Humaira Khan, Robert Macdonald, Ciara Mackenzie, Julia Maddison, Tarang Majmudar, Vivek Malhotra, Ranjit Manchanda, Roger Moshy, Hans Nagar, Adam Nahkretski, Julia Palmer, Robert Parker, Selvi Radhikavikram, Harinder Rai, Bruce Ramsay, Natalia Rosello, Michelle Russell, Ahmad Sayasneh, Partha Sengupta, Aarti Sharma, Anju Sinha, Lavanya Vitta,, Mark Willett, Nicholas Wood, Ahmed Darwish

**Affiliations:** 1 PanBirmingham Gynaecological Cancer Centre, Sandwell and West Birmingham Hospitals NHS Trust, Birmingham B71 4HJ, UK; 2 Department of Cancer and Genomic Sciences, University of Birmingham, Birmingham B71 4HJ, UK; 3 Department of Applied Health Science, University of Birmingham, Birmingham, UK; 4 NIHR Birmingham Biomedical Research Centre, University Hospitals Birmingham NHS Foundation Trust and University of Birmingham, Birmingham, UK; 5 Department of Development and Regeneration, KU Leuven, Leuven, Belgium; 6 Leuven Unit for Health Technology Assessment Research (LUHTAR), KU Leuven, Leuven, Belgium; 7 Southampton University Hospitals, NHS Trust, Southampton, UK; 8 Durham and Darlington NHS Trust, Darlington, UK; 9 West Hertfordshire Hospitals NHS Trust, Watford, UK; 10 Centre for Medical Imaging, University College London, London, UK; 11 University of Nottingham, Nottingham, UK; 12 St Peter's College, University of Oxford, Oxford, UK; 13 Department of Obstetrics and Gynaecology, University Hospitals KU Leuven, Leuven, Belgium; 14 Faculty of Medicine, Department of Metabolism, Digestion and Reproduction, Imperial College London, London, UK; 15 Patient Representative, Birmingham, UK; 16 University of Exeter Medical School, University of Exeter, Exeter, UK; 17 Department of Women’s Cancer, Elizabeth Garrett Anderson Institute for Women’s Health, University College London, London, UK; 18 MRC Clinical Trials Unit, Institute of Clinical Trials and Methodology, University College London, London, UK; 19 Birmingham Clinical Trials Unit, University of Birmingham, UK

## Abstract

**Objective:**

To investigate the accuracy of risk prediction models and scores for diagnosing ovarian cancer in premenopausal women presenting to secondary care with symptoms and abnormal test results.

**Design:**

Prospective cohort study.

**Setting:**

Secondary care in 23 hospitals in the UK between June 2015 and March 2023.

**Participants:**

Premenopausal women presenting with non-specific symptoms, and raised serum levels of cancer antigen 125 or abnormal imaging results, were prospectively recruited, predominantly referred through the NHS urgent suspected cancer pathway from primary care. A head-to-head comparison of the accuracy of the six risk prediction models and scores was conducted using donated blood and ultrasound scans performed by NHS staff trained in the use of International Ovarian Tumour Analysis (IOTA) imaging terminology. The index tests used were Risk of Malignancy Index 1 (with pre-stated thresholds of 200, 250), Risk of Malignancy Algorithm (7.4%, 11.4%, 12.5%, 13.1%), IOTA Assessment of Different Neoplasias in the adnEXa (ADNEX) (3%, 10%), IOTA simple rules risk model (3%, 10%), IOTA simple rules, and cancer antigen 125 (CA 125, 87 IU/mL). Participants were classified as having primary invasive ovarian cancer versus having benign or normal pathology according to the reference standard determined from surgical specimens or biopsies by histology or cytology, if undertaken, or else at 12 month follow-up. After June 2018, because of covid restrictions and concerns about sample size, recruitment was restricted to only women undergoing surgery within three months of presentation to clinic (in whom ovarian cancer was more likely).

**Main outcome measures:**

Diagnostic accuracy at predicting primary invasive ovarian cancer versus benign or normal histology, assessed by analysing the sensitivity, specificity, C index, area under receiver operating characteristic curve, positive and negative predictive values, and calibration plots in participants with conclusive reference standard results and available index test data.

**Results:**

88 of 1211 premenopausal women received diagnoses of primary ovarian cancer: 49 of 857 women in the pre-June 2018 cohort (prevalence of 5.7%) and 39 of 354 women in the post-June 2018 cohort (11.0%). For the diagnosis of primary ovarian cancer (n=799 women, after exclusion of 58 other diagnoses), Risk of Malignancy Index 1 at the 250 threshold had a sensitivity of 42.6% (95% confidence interval (CI) 28.3 to 57.8; specificity 96.5%, 94.7 to 97.8). Compared with Risk of Malignancy Index 1 at the 250 threshold, CA 125 and all other tests had higher sensitivity (CA 125 at 87 IU/mL threshold: 55.1%, 40.2 to 69.3, P=0.06; Risk of Malignancy Algorithm at 11.4% threshold: 79.2%, 65.0 to 89.5, P<0.001; IOTA ADNEX at 10% threshold: 89.1%, 76.4 to 96.4, P<0.001; IOTA simple rules risk at 10% threshold: 83.0%, 69.2 to 92.4, P<0.001; IOTA simple rules: 75.0%, 56.6 to 88.5, P=0.01) and lower specificity (CA 125 at 87 IU/mL threshold: 89.0%, 86.5 to 91.2, P<0.001; Risk of Malignancy Algorithm at 11.4% threshold: 73.1%, 69.6 to 76.3, P<0.001; IOTA ADNEX at 10% threshold: 75.1%, 71.4 to 78.6, P<0.001; IOTA simple rules risk at 10% threshold: 76.0%, 72.4 to 79.3, P<0.001; IOTA simple rules: 95.2%, 93.0 to 96.9, P=0.06). Results for IOTA simple rules were inconclusive in 120 of 799 participants. Analysis of the complete cohort (n=1211), including the 354 premenopausal women with a higher likelihood of developing ovarian cancer, yielded similar results.

**Conclusions:**

Compared to Risk of Malignancy Index 1 at 250 threshold—the test currently used in NHS secondary care to triage women to tertiary care—most tests improve sensitivity but reduce specificity. Ultrasound triage with the IOTA ADNEX model at 10% in secondary care demonstrated the highest sensitivity gain, with a comparable decline in specificity to other comparator tests. Ultrasound with the IOTA ADNEX model at 10% should be considered the new standard of care test for triaging premenopausal women in secondary care. Implementation should incorporate staff training and quality assurance.

**Trial registration:**

ISRCTN17160843.

## Introduction

Ovarian cancer is a challenging disease to diagnose, with patients typically visiting general practitioners (GPs) or primary care multiple times before testing is initiated.^1^ ^2^ Currently, most women receive a cancer diagnosis at an advanced stage because of non-specific symptoms and suboptimal diagnostic pathways. Diagnosis is especially challenging in premenopausal women due to low ovarian cancer prevalence (approximately 93 000 women younger than 49 years receive diagnoses globally each year, age standardised rate of 2.2-3.6/100 000^3^), non-specific symptoms, non-specifically elevated cancer antigen 125 (CA 125; eg, during menstruation) and physiological ovarian cysts on ultrasound (as a result of ovarian cysts being frequently present in premenopausal women). In England and Wales, 40% of women with ovarian cancer are admitted to hospital as an emergency four weeks before receiving an ovarian cancer diagnosis and are five times more likely to die within six months than women referred through urgent suspected cancer pathways.^4^ Ovarian cancer survival in the UK is substantially lower than in other western countries.

The National Institute for Health and Care Excellence (NICE) guidelines recommend sequential testing using serum CA 125 and pelvic ultrasound for women presenting to their GP with symptoms such as persistent abdominal distension, feeling full, pelvic pain, increased urinary urgency, unexplained weight loss, fatigue, or changes in bowel habit.^5^ Women with elevated CA 125 serum levels or ultrasound findings considered abnormal in primary care are referred to gynaecologists in secondary care hospitals through the urgent suspected cancer pathway in the NHS: patients referred to hospital receive a cancer/non-cancer diagnosis within 28 days of referral, and patients with a diagnosis of cancer receive first treatment within 62 days of referral.^6^ ^7^

**Fig 1:**
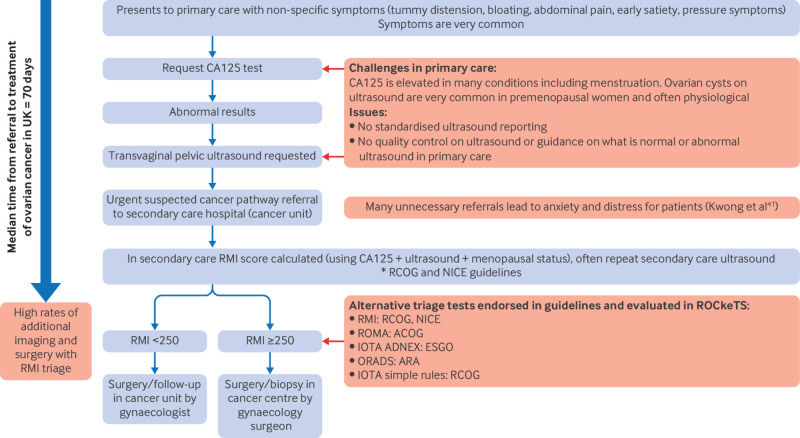
Flowchart of referral pathways for patients presenting with non-specific symptoms of ovarian cancer to primary care in the UK. CA 125=cancer antigen 125; RMI=Risk of Malignancy Index; ROMA=Risk of Malignancy Algorithm; RCOG=Royal College of Obstetrics and Gynaecology; ACOG=American College of Obstetrics and Gynecology; IOTA=International Ovarian Tumour Analysis; ADNEX=Assessment of Different Neoplasias in the Adnexa; ESGO=European Society of Gynaecological Oncology; ORADS= Ovarian-Adnexal Reporting and Data System; ARA=American Radiology Association

Poor performance of current diagnostic testing contributes to the challenge of timely, accurate diagnosis: only 50% of patients who have early stage ovarian cancer can be identified with CA 125 test results. CA 125 can be elevated as a result of other benign conditions, while ultrasonography in primary care lacks standardisation or quality control and is associated with long waiting times.^8^ ^9^ NICE guidance recommends the risk of malignancy index algorithm to triage women referred with suspected ovarian cancer in hospital, using age and menopausal status to generate a risk score (known as the risk of malignancy index score).^5^ ^10^ Women with a risk of malignancy index score ≥250 are referred to tertiary care hospitals (gynaecological cancer centres) for gynaecological cancer surgery, while those with a risk of malignancy index <250 are managed in the referring secondary care hospital with surgery or surveillance by gynaecologists (fig 1). Women who are referred with the current pathway experience high rates of surgery and additional imaging (magnetic resonance imaging (MRI) or computed tomography (CT)).^11^

Accurate triage is important: triaging high risk for ovarian cancer preoperatively enables appropriate surgery at first attempt in a tertiary, specialist cancer centre, which improves patient survival.^5^ ^12^ ^13^ ^14^ Improving diagnostic pathways for premenopausal women is a key unmet need. Alternative risk prediction models to the Risk of Malignancy Index 1 250 endorsed by professional societies include the Risk of Malignancy Algorithm (combining human epididymis protein 4 and CA 125 biomarkers, stratified by menopausal status), and several ultrasound based models (the Ovarian-Adnexal Reporting and Data System, the IOTA Assessment of Different Neoplasias in the Adnexa (ADNEX), and IOTA simple rules).^15^ ^16^ ^17^ ^18^ ^19^ ^20^ ^21^

A 2022 Cochrane systematic review highlighted the absence of high quality, head-to-head comparative test accuracy studies which included studies conducted in high prevalence settings and were applicable to a primary care referred population (16-27%).^22^ The Cochrane review also demonstrated variation in performance of risk prediction models between premenopausal and postmenopausal women, reflecting differences in disease prevalence and histology range spectrum.^22^ Additionally, the trade-offs between identifying true positives (enabling early diagnosis and better survival) versus limiting false positives (reducing unnecessary referral, patient anxiety, and surgery) are different for premenopausal women compared to those for postmenopausal women, where preserving fertility and ovarian function are critical considerations in premenopausal women**.**

Our ROCkeTS study aimed to improve on existing evidence by identifying the best diagnostic test for women referred to secondary care hospitals with symptoms of ovarian cancer and abnormal CA 125 test results or ultrasound results. We evaluated the accuracy of alternative risk prediction models—IOTA simple rules, IOTA simple rules risk model, Risk of Malignancy Algorithm, Ovarian-Adnexal Reporting and Data System, and IOTA ADNEX—compared to the Risk of Malignancy Index 1 at the 250 threshold standard of care triage test in the UK (box 1, supplementary appendix 1) against a reference standard of histology from surgery or biopsy or follow-up. Results for postmenopausal women have been previously reported.^23^

Box 1 **Risk prediction models and scores evaluated in the ROCkeTS study (to determine accuracy of diagnostic tests for ovarian cancer in premenopausal women)**

**Risk of Malignancy Index 1**

Combines menopausal status, CA 125 tumour marker levels, and specific ultrasound features to provide a numerical score. Ultrasound features are scored as per a U score, wherein the ultrasound result is scored with one point for each of the following characteristics: multilocular cysts, solid areas, metastases, ascites, and bilateral lesions. U=0 (for an ultrasound score of 0), U=1 (for an ultrasound score of 1), U=3 (for an ultrasound score of 2 to 5). Commonly used cut-off thresholds for this model are 200 and 250 to guide patient management

**Risk of Malignancy Algorithm**

Combines serum levels of two tumour markers, human epididymis protein 4 and CA 125, in an algorithm to provide a numerical score. Thresholds vary by manufacturer and by menopausal status of the patient

**International Ovarian Tumour Analysis (IOTA) simple rules**

Ultrasound based classifier based on five benign and five malignant ultrasound features. Results are expressed as benign, malignant, or inconclusive

**IOT Assessment of Different Neoplasias in the Adnexa (ADNEX)**

Risk prediction model combining three clinical variables and six ultrasound variables. The model expresses risk in per cent for borderline, stage 1 cancer, stage 2-4 cancer, and secondary metastatic cancer; 3% and 10% are commonly used threshold values

**IOTA simple rules risk model**

A logistic regression model that uses the 10 ultrasound features in IOTA simple rules, plus the type of centre (oncology centre or non-oncology centre) as predictors to calculate the likelihood of malignancy in adnexal masses

**Cancer antigen 125**

Serum level of tumour marker CA 125 in IU/mL

**Ovarian-Adnexal Reporting and Data System**

A clinical support system that provides a standardised lexicon for describing ovarian and adnexal lesions and stratifies them using a numerical score based on morphological features to indicate risk of malignancy (eg, 0-3 (normal, benign, or low risk) *v* 4-5 (equivalent to 10% (intermediate or high) risk of malignancy))

## Methods

Our report adheres to the STARD and TRIPOD checklists (supplementary appendix 1, supplementary tables 10-11).^24^ ^25^ The trial protocol is available at https://www.birmingham.ac.uk/research/bctu/trials/pd/rockets and has previously been published.^26^ Methods for ROCkeTS has been previously described and in the statistical analysis plan (supplementary appendix 1).

### Participants

We consecutively recruited women aged 16-90 years who were newly presenting to hospital and were referred to hospitals within the UK. Research nurses from the UK National Cancer Research Network screened patients attending hospital clinics referred from primary care through urgent suspected cancer pathways, ultrasound clinics, gynaecology clinics, or presenting through emergency admissions. Eligible women had symptoms consistent with NICE guidance for ovarian cancer (including, but not restricted to, persistent or frequent abdominal distension, feeling full (early satiety) or loss of appetite, pelvic or abdominal pain, and increased urinary urgency and/or frequency^5^) as well as elevated CA 125 levels or abnormal ultrasound findings. At recruitment, participants self-reported sex and ethnicity and completed a structured questionnaire to obtain medical and gynaecological history. Exclusion criteria were pregnancy, existing non-ovarian malignancy, and previous ovarian malignancy.

Participants were categorised as premenopausal or postmenopausal at recruitment by their age (<50, ≥51 years) and whether they had menstruated in the past 12 months. After recruitment, participating perimenopausal women were re-categorised based on a self-reported history of vaginal bleeding to allow analysis with existing risk prediction models that incorporate different thresholds or covariates based on menopausal status. 

The study protocol underwent substantial evolution from initial publication.^26^ Our study initially consecutively recruited eligible women who were scheduled for surgical intervention within three months of referral to hospital or who were being managed through conservative approaches, including surveillance or discharge from hospital as clinically indicated, with planned wellbeing assessments based on questionnaires at 12 month follow-up.

At an interim analysis, we observed ovarian cancer prevalence to be 3%, which is sufficient for estimating specificity but inadequate to estimate comparisons of sensitivity between tests and models. Consequently, we introduced adaptations to the protocol in March 2018, excluding women with simple ovarian cysts <5 cm and normal CA 125 levels because of their very low cancer risk, and from June 2018, recruitment focused solely on premenopausal women who were newly presenting to clinic and were scheduled for surgery within three months. From June 2018, undergoing IOTA ultrasound scan was deemed optional, acknowledging both the scheduling challenges for women undergoing surgery for suspected cancer and ethical concerns about requiring additional hospital visits during the covid-19 pandemic (from 2020 to 2022).

The study cohort comprised two groups: the pre-protocol change cohort (cohort 1), which recruited presurgical and conservatively managed patients until June 2018; and the post-protocol change cohort, which recruited participants after June 2018 and comprised only pre-surgical patients (cohort 2). We performed a sensitivity analysis to account for potential changes in spectrum, with the pre-protocol change cohort seen as most likely to be representative and was therefore used in the primary analysis.

Three eligible patients were excluded after recruitment: one had an unexpected pregnancy, one had a history of non-ovarian malignancy, and one declined surgery. All eligible and willing patients were included. Investigators used the current NHS standard of care triage test, Risk of Malignancy Index 1 at 250 threshold, to manage patients.

### Index tests

All patients completed a symptom questionnaire, donated a blood sample, and underwent transabdominal and transvaginal ultrasound scans. Serum samples were collected, processed, and stored according to predefined standard operating procedures, consistent with consensus guidance from the Early Detection Research Network (supplementary appendix 1).^27^ Samples were transported and stored at −80°C until analysis at NHS South Tyne and Wear Pathology Services laboratories. For consistent analyses, samples were thawed in batches and tested for CA 125 and human epididymis protein 4 using Roche Cobas e802 modules. Human epididymis protein 4 and cancer antigen 125 measurements used electrochemiluminescence immunoassay technology, adhering to manufacturer recommendations. Roche Elecsys assay kits were obtained from Roche Diagnostics.

We evaluated the following index tests (box 1, supplementary appendix 1): Risk of Malignancy Algorithm, (which combines CA 125 and human epididymis protein 4 tumour markers) in an algorithm at the manufacturer recommended threshold of 11.4% for premenopausal women and at previously reported thresholds of 7.4%, 12.5%, 13.1%^28^; Risk of Malignancy Index 1 (which measures CA 125 and some ultrasound features) at thresholds of 200 and 250 (the 250 threshold—the current standard of care diagnostic approach for triaging patients to gynaecological cancer centres in the NHS—was chosen as the comparator test)^5^ ^18^; serum CA 125 measurement at a threshold of 87 IU/mL based on its association with a positive predictive value of 3% in primary care as detailed by Funston et al.^29^

We also evaluated ultrasound based tests. Three tests developed by the IOTA consortium were evaluated: two models (IOTA ADNEX and the IOTA simple rules risk model at a primary thresholds of 10% and secondary thresholds of 3%) and one classifier (IOTA simple rules).^17^ ^20^ ^30^ ^31^ ^32^ IOTA ADNEX is now considered a medical device and is manufactured by Gynaia.^33^ We evaluated the Ovarian-Adnexal Reporting and Data System in a post hoc analysis using IOTA variables from the ROCkeTS ultrasound case report form, retrospectively mapped to the Ovarian-Adnexal Reporting and Data System lexicon 1-3 versus 4-5 using previously described methodological approaches.^20^ ^34^

We define IOTA ultrasound scan as one wherein IOTA terminology—a set of terms, definitions, and measurements used to precisely describe adnexal mass characteristics found in ultrasound scans—has been used to describe the ultrasound findings.^35^ Sonographers who had undertaken comprehensive training in IOTA terminology (including one day, in-person training as well as online instruction and a formal exam) conducted ultrasound tests in our study. A mandatory quality assessment process was implemented, with a sample of ultrasound images and reports centrally reviewed by the IOTA team of ultrasound experts, as described previously.^23^ No minimum ultrasound experience requirement was stipulated. Scans were primarily performed by level 2 (non-medical) sonographers, which mirrors real world clinical practice, where sonographers with varying levels of experience perform pelvic ultrasound scans.

All diagnostic tests and subsequent surgical interventions or biopsies were required to be completed within three months of patient recruitment. All tests were conducted blinded to the reference standard.

### Reference standard

For cohort 1, we used histology of surgical specimens, biopsies, or cytology or 12 month surveillance for women who did not undergo surgery as the reference standard. In cohort 2, study participation ended at surgery, biopsy, or cytology, which served as the reference standard.

Pathology data were sourced from specialist gynaecological pathology reports from the 40 cancer centres in the UK, where treatment plans for women undergoing surgery for suspected ovarian cancer are discussed at specialist gynaecological oncology multidisciplinary team meetings.

For participants under surveillance, we ascertained wellbeing at 12 months using postal questionnaires completed by the patient and research questionnaires completed by nurses using hospital records from a clinic visit or by contacting patients by telephone. No diagnosis of cancer or histology results were based on self-reported questionnaires alone. Both information sources were cross referenced to identify any cancer diagnoses received by the patient within 12 months of study recruitment.

Reference standard results were not available to sonographers or the research team before trial entry, although clinical information was available as this is considered usual clinical practice.

### Outcomes

The primary outcome focused on the diagnostic accuracy of index tests in identifying ovarian cancer. We defined accuracy as a binary outcome: primary invasive malignant neoplasms diagnosed through surgical or biopsy histology, versus benign, normal, or surveillance findings. The definition of primary invasive ovarian cancer included ovarian, fallopian tube, and primary peritoneal cancers.

The secondary outcome included a broader spectrum of malignancies: primary invasive cancers, secondary malignant neoplasms metastatic to the ovary, borderline neoplasms, and neoplasms of uncertain or unknown behaviour diagnosed through surgery, biopsy, or cytology, versus benign or normal follow-up findings. We also analysed the secondary outcome with borderline ovarian tumours grouped with benign or normal follow-up findings rather than with malignancies. 

### Statistical analysis

We entered clinical study data in structured case report forms on an electronic study management platform hosted by MedSciNet (https://rockets.medscinet.com/). Data were entered by research nurses at study sites. Trial statisticians at the Birmingham Clinical Trials Unit cleaned the data, and resolved data queries (supplementary appendix 1 includes the statistical analysis plan). Diagnostic accuracy was assessed using sensitivity, specificity, and the positive and negative predictive values; risk prediction tools were dichotomised at different thresholds. We assessed the difference in sensitivity and specificity (and their corresponding 95% confidence intervals (CIs)) comparing tests using the exact McNemar’s test with asymptotic CIs. Multiple testing was accounted for by use of the Bonferroni correction (11 pairwise comparisons, P=0.005).^22^ ^23^ We designated no single measure of accuracy as the primary endpoint a priori, to fully evaluate the trade-offs inherent in the performance of diagnostic tests.

We further assessed global accuracy performance in terms of discrimination (using a C index and receiver operating characteristic plot) and calibration (using calibration plots and calibration slope). We used the pmcalplot command in Stata to generate the calibration plots.^36^ When index tests produced inconclusive results (IOTA simple rules), we opted not to classify inconclusive results as positive, as this could have led to an overestimation of test performance for sensitivity estimates.

Women with missing index test data were excluded from both primary and secondary analyses. Participants with missing or inconclusive reference standard results were excluded from the primary outcome definition (presence or absence of ovarian cancer) but were included in the secondary outcome (presence of any cancer) to make the best use of participant data.

We conducted sensitivity analyses for the primary and secondary definitions of ovarian cancer when missing index test results were imputed. We used the multiple imputation by chained equations for predictors of index test combinations by replacing missing values with plausible values based on the distribution of the observed data.^37^ Multiple imputation was performed using the mi package in Stata 17.^38^

We performed initial analysis for primary and secondary outcomes in the pre-protocol change cohort, as well as in a combined cohort comprising participants from cohorts 1 and 2.

#### Sample size

The original sample size was based on local audit data (unpublished) to estimate the performance of the Risk of Malignancy Index 1 in predicting risk of ovarian cancer in premenopausal women, because earlier systematic reviews did not provide separate estimates for premenopausal and postmenopausal women. Based on the performance of Risk of Malignancy Index 1 having a sensitivity of 72% and specificity of 46%, the study was designed to detect 10% increases in sensitivity and in specificity, assuming a 10% prevalence of ovarian cancer in premenopausal women referred to secondary care.^39^ A sample size of 1000 participants would provide 100 ovarian cancer events in which to build new models combining symptom and test data (adequate events to model 10 predictor variables) and will provide 90% power to detect an increase in specificity of 8% (from 46% for Risk of Malignancy Index 1 to 54%). With a predicted loss to follow-up of up to 5%, the final sample size required is 1050 women.^40^

A 2018 interim analysis of prevalence observed a much lower prevalence (3%) in premenopausal women than previously assumed (10%). Therefore, both sample size and inclusion criteria were adapted to ensure that the study had adequate power to estimate the difference in sensitivities of the index tests by recruiting 105 women (accounting for 5% dropout) identified as having ovarian cancer. Following the change of the inclusion criteria, while a prevalence of 14.9% was expected in women who undergo surgery, the revised sample size of 880 patients was based on the assumption that only 11.9% (80%) would actually have surgery.

### Patient and public involvement

The study was supported by a patient co-applicant who had a crucial role throughout the research process. HS provided regular input at key stages of the study: application for funding, study design, participant recruitment, and dissemination of findings. Patient advocates from Target Ovarian Cancer—a charity that works to save lives of people with ovarian cancer by improving early diagnosis and increasing symptom awareness—partnered with the research team to shape the study design, to develop and review informational materials for the study, and to assess the burden of questionnaires from the patient’s perspective.

At the conclusion of the study, HS and Target Ovarian Cancer contributed feedback on the findings and gave valuable input into the interpretation of results and outcome measures, ensuring that the findings were meaningful from a patient centred perspective.

## Results

### Characteristics of study population

**Fig 2:**
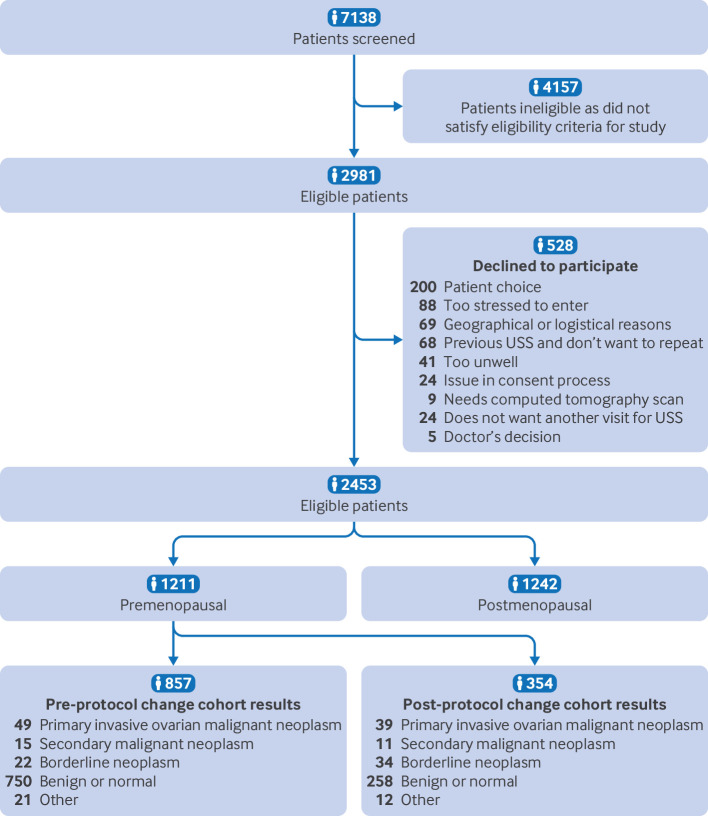
Recruitment flow of patients in the ROCkeTS study to determine accuracy of diagnostic tests for ovarian cancer in premenopausal women with non-specific symptoms and abnormal test results. USS=ultrasound scan

We recruited 2453 eligible premenopausal and postmenopausal women referred to 23 UK hospitals between 30 June 2015 and 23 March 2023, and followed them up to 31 March 2023 (fig 2). Results of the 1242 women in the postmenopausal cohort have been reported separately.^23^ We report our findings from a cohort of 1211 premenopausal participants, comprising 857 women recruited up to June 2018 under the initial protocol (cohort 1) and 354 women recruited under the amended protocol restricted to women scheduled for surgery within three months of recruitment (cohort 2). Most of the participants in cohort 1 were recruited from primary to secondary care through the urgent suspected cancer pathway (n=574, 67%). The main analysis focuses on the results of cohort 1, because it represents real world practice and has low missingness of IOTA ultrasound scans. Findings from participants in cohort 2 are included in a sensitivity analysis in a combined cohort analysis.

Women in cohort 1 had a median age of 44.1 (interquartile range (IQR) 35.0-48.7) years. Most women were white (n=732, 85.4%), had never smoked (n=476, 55.5%), and were not using contraception (n=502, 58.6%) (table 1). More than half of the women (n=444, 51.8%) had comorbidities: 137 (16.0%) had fibroids, 135 (15.8%) had endometriosis, and 121 (14.1%) had irritable bowel syndrome. Some participants (n=184, 21%) had undergone previous surgery: 44 (5.1%) had a hysterectomy, 82 (9.6%) had a cystectomy, 33 (3.9%) had a salpingectomy, and 25 (2.9%) had an oophorectomy. More than a quarter of women in cohort 1 (n=204, 24%) reported a family history of cancer: 42 (4.9%) reported ovarian cancer, 98 (11.4%) reported breast cancer, 48 (5.6%) reported colon cancer and 16 (1.9%) reported uterine cancer (table 1). We did not observe any systematic differences in patient characteristics between cohort 1 and cohort 2.

**Table 1:** Characteristics, medical conditions, and clinical history of participants recruited to ROCkeTS study to determine accuracy of diagnostic tests for ovarian cancer in premenopausal women with non-specific symptoms and abnormal test results. Data are number (%) of participants unless otherwise specified

**Characteristics**	Cohort 1 (n=857)*	**Cohort 2 (n=354)†**	**Total (n=1211)**
**Participant characteristics**			
Median (IQR) age (years)	44.1 (35.0-48.7)	40.2 (31.1-47.1)	43.2 (33.7-48.3)
Median (IQR) height (cm)	164.0 (159.0-168.0)	164 (160.0-169.0)	164 (160-169.0)
Median (IQR) weight (kg)	71.6 (62.0-85.4)	76 (64.0-90.0)	73 (62.0-86.0)
Ethnicity:			
White	732 (85.4)	294 (83.1)	1026 (84.7)
Mixed	18 (2.1)	9 (2.5)	27 (2.2)
Asian	51 (5.9)	22 (6.4)	73 (6.0)
Black	20 (2.3)	10 (2.8)	30 (2.5)
Other	10 (1.2)	4 (1.1)	14 (1.2)
Never smoked	476 (55.5)	211 (59.6)	687 (56.7)
Median (IQR) No of units of alcohol consumed per week	2 (0-9)	2 (0-6)	2 (0-8)
**Current medical conditions‡**			
None	413 (48.2)	187 (52.8)	600 (49.6)
Fibroids	137 (16.0)	47 (13.3)	184 (15.2)
Endometriosis	135 (15.8)	34 (9.6)	169 (14.0)
Irritable bowel syndrome	121 (14.1)	35 (9.9)	156 (12.9)
Hypertension	66 (7.7)	26 (7.3)	92 (7.6)
Arthritis	59 (6.9)	22 (6.2)	81 (6.7)
Adhesions	34 (4.0)	11 (3.1)	45 (3.7)
Diabetes	22 (2.6)	10 (2.8)	32 (2.6)
Uterine polyps	25 (2.9)	4 (1.1)	29 (2.4)
Uterine or bladder prolapse	20 (2.3)	7 (2.0)	27 (2.2)
High cholesterol	21 (2.5)	11 (3.1)	32 (2.6)
**Medical history**			
Surgical history			
Hysterectomy	44 (5.1)	8 (2.3)	52 (4.3)
Ovarian cystectomy	82 (9.6)	34 (9.6)	116 (9.6)
Salpingectomy	33 (3.9)	13 (3.7)	46 (3.8)
Oophorectomy	25 (2.9)	6 (1.7)	31 (2.6)
Previous diagnosis of cancer			
Breast	15 (1.8)	3 (0.9)	18 (1.5)
Other	20 (2.3)	7 (2.0)	27 (2.2)
Family cancer history			
Ovary	42 (4.9)	14 (4.0)	56 (4.6)
Breast	98 (11.4)	38 (10.7)	136 (11.2)
Colon	48 (5.6)	27 (7.6)	75 (6.2)
Uterus	16 (1.9)	9 (2.5)	25 (2.1)
Stated sexually active	604 (70.5)	238 (67.2)	842 (69.5)
No of pregnancies	2 (0-3)	1 (0-3)	1 (0-3)
No of live births§	2 (1-2)	2 (1-3)	2 (1-3)
No of vaginal deliveries§	2 (1-2)	1 (0-2)	1 (1-2)
No of caesarean sections§	0 (0-1)	0 (0-1)	0 (0-1)
Trying to get pregnant	70 (8.2)	20 (5.7)	90 (7.4)
History of subfertility	116 (13.5)	45 (12.7)	161 (13.3)
History of ovarian stimulation	49 (5.7)	19 (5.4)	68 (5.6)
Painful periods	471 (55.0)	205 (57.9)	676 (55.8)
Change in the nature of periods	409 (47.7)	170 (48.0)	579 (47.8)
Change in period pain in past year	350 (40.8)	138 (39.0)	488 (40.3)
Irregular periods in past year	389 (45.4)	152 (42.9)	541 (44.7)
Heavy periods in past year	412 (48.1)	172 (48.6)	584 (48.2)
Currently using contraception	226 (31.0)	117 (37.0)	345 (33.0)

Data completeness is 100% for age, current conditions, and medical history; between 96% and 97% for other characteristics, report of sexual activity, and gravidity; between 86% and 87% for gynaecological history.

* Participants recruited up until June 2018(including pre-surgical and conservatively managed patients).

† Participants recruited after June 2018 (after the study protocol adaptation).

‡ Current conditions with fewer than 20 cases (<2%) were reported in women with adenomyosis, diverticulitis, sexually transmitted infection, epilepsy, heart disease, pelvic inflammatory disease, vulva pain/vulvodynia, and jaundice.

§ Live births, vaginal deliveries, and caesarean sections were counted in participants with one or more pregnancies.

### Prevalence of ovarian cancer

Eighty eight (7.3%) of the 1211 women across both cohorts received a diagnosis of primary invasive ovarian cancer, the primary outcome of our study: 47 women (53.4%) received diagnoses at International Federation of Gynaecology and Obstetrics (FIGO) stage 1, six (6.9%) at stage 2, 24 (27.2%) at stage 3, and one (1.1%) at stage 4 (stage data were missing for 10 participants). Five women received their diagnoses during the 12 month follow-up period, 83 from surgery or biopsy histology results (73 (88%) of which had epithelial histology types; table 2).

**Table 2:** Histopathology of pelvic masses in women recruited to the ROCkeTS study (to determine accuracy of diagnostic tests for ovarian cancer in premenopausal women with non-specific symptoms and abnormal test results) who underwent surgery and/or biopsy (n=837) or were identified by follow-up (n=5) in the combined cohort. Data are number (%) of participants unless otherwise specified

Histology result	No of participants receiving diagnosis across the combined cohort n=842)
Primary invasive malignant neoplasm	88 (10.5)
From surgery and/or biopsy	
Malignant epithelial	
Clear cell	7
Endometrioid	14
High grade serous	20
Low grade serous	9
Mucinous	21
Undifferentiated	1
Not available	1
Germ cell	
Immature teratoma	1
Yolk sac tumour	1
Sex cord	
Granulosa cell tumour	5
Not reported	1
Unavailable	2
Diagnosis of ovarian cancer given during follow-up period (not reported*)	5
Secondary malignant neoplasm	21 (2.5)
Endometrium	11
Appendix	5
Colon	2
Stomach	2
Breast	1
Borderline	58 (6.9)
Mucinous	24
Serous	14
Seromucinous	1
Serous and mucinous	1
Not available	18
Benign	601 (71.4)
Benign tumours	
Serous adenofibroma	4
Serous cystadenoma and fibroma	99
Mucinous adenofibroma	2
Mucinous cystadenomas	109
Mature teratoma	116
Brenner tumour	2
Endometrioma	142
Fibroma/fibrothecoma	8
Functional	37
Other pathology	
Endometrioid adenofibroma	8
Uterine fibroid	29
Chronic inflammation/abscess	13
Hydrosalpinx	7
Struma ovarii	6
Seromucinous cystadenoma	3
Mature teratoma and mucinous cystadenoma	1
Mucinous cystadenoma and Brenner tumour	2
Not available	13
Normal	47 (5.6)
No histology	27 (3.2)

* For five patients, histology results were classified as having ovarian cancer on the basis of free text information, but did not have structured histology details recorded. For these women, a diagnostic category of “other” was selected, with free text entries describing malignancy consistent with ovarian cancer (eg, high grade serous carcinoma cytokeratin 7, Wilms tumour 1, cancer antigen 125, oestrogen receptor positive, cytokeratin 20 negative; poorly differentiated cancer—signet ring; the features within the left ovary are of a serous papillary). However, no additional structured fields were completed to confirm the final histological diagnosis.

Forty nine (5.7%) of the 857 women in cohort 1 received a diagnosis of ovarian cancer. Fifty eight (6.8%) women were reported as “other” and were excluded from estimation of accuracy for the primary outcome (13 had a missing primary outcome, 22 had borderline neoplasm, seven had no histology, 10 had secondary malignant neoplasm, one had a diagnostic category of “other,” and five reported a diagnosis of non-ovarian cancer at 12 month follow-up).

### Accuracy of index tests

In cohort 1, 581 (72.7%) of 799 women included in the analysis for the primary ovarian cancer classification had complete data for all index tests. Results were available for CA 125 for 795 (99.5%) women, Risk of Malignancy Algorithm for 750 (93.9%), Risk of Malignancy Index 1 for 672 (84.1%), IOTA simple rules risk model for 668 (83.6%), and IOTA ADNEX for 617 (77.2%) (table 3, supplementary appendix 1, supplementary table 1). For the IOTA simple rules, results were only available for 553 (69.2%) women, as 126 women had missing results and 120 had inconclusive results.

**Table 3:** Diagnostic performance statistics of index test combinations for identifying primary ovarian cancer in women recruited to the ROCkeTS study between June 2015 and June 2018 (cohort 1)

Index test combination	Threshold	Diagnosis based on reference standard (n=799; No (%) of participants)		No (%) of participants	Sensitivity (%; 95% CI)	Specificity (%; 95% CI)	C index (AUC) (95% CI)	Positive predictive value (%) (95% CI)	Negative predictive value (%) (95% CI)	Pairwise comparison with RMI 1 (at 250 threshold) (%; 95% CI; P value)		
		OC (n=49)	No OC (n=750)							Sensitivity	Specificity	No of participants
RMI 1	Missing	2 (4.1)	125 (16.7)	—	—	—	—	—	—	—	—	—
	>200	23 (46.9)	29 (3.9)	672 (84.1)	48.9 (34.1 to 63.9)	95.4 (93.4 to 96.9)	0.85 (0.79 to 0.91)	44.2 (30.5 to 58.7)	96.1 (94.3 to 97.5)	−6.4 (−15.5 to 2.7), P=0.25	1.1 (0.1 to 2.1), P=0.02	672
	<200	24 (49.0)	596 (79.5)									
	>250	20 (40.8)	22 (2.9)		42.6 (28.3 to 57.8)	96.5 (94.7 to 97.8)		47.6 (32.0 to 63.6)	95.7 (93.8 to 97.2)	—	—	—
	<250	27 (55.1)	603 (80.4)									
ROMA	Missing	1 (2.0)	48 (6.4)	—	—	—	—	—	—	—	—	—
	>7.4%	43 (87.8)	372 (49.6)	750 (93.9)	89.6 (77.3 to 96.5)	47.0 (43.3 to 50.8)	0.84 (0.77 to 0.92)	10.4 (7.6 to 13.7)	98.5 (96.6 to 99.5)	−47.8 (−64.4 to −31.2), P<0.001	49.3 (45.0 to 53.7), P<0.001	636
	<7.4%	5 (10.2)	330 (44.0)									
	>11.4%	38 (77.6)	189 (25.2)		79.2 (65.0 to 89.5)	73.1 (69.6 to 76.3)		16.7 (12.1 to 22.2)	98.1 (96.5 to 99.1)	−37.0 (−53.1 to −20.8), P<0.001	23.6 (19.6 to 27.5), P<0.001	636
	<11.4%	10 (20.4)	513 (68.4)									
	>12.5%	37 (75.5)	164 (21.9)		77.1 (62.7 to 88.0)	68.6 (64.4 to 72.5)		18.4 (13.3 to 24.5)	97.0 (94.7 to 98.5)	−34.8 (−50.7 to −18.8), P<0.001	19.8 (16.0 to 23.6), P<0.001	636
	<12.5%	11 (22.4)	358 (47.7)									
	>13.1%	36 (73.5)	149 (19.9)		75.0 (60.4 to 86.4)	78.8 (75.6 to 81.7)		19.5 (14.0 to 25.9)	97.9 (96.3 to 98.9)	−32.6 (−48.3 to −16.9), P<0.001	17.6 (13.9 to 21.3), P<0.001	636
	<13.1%	12 (24.5)	553 (73.7)									
IOTA ADNEX	Missing	3 (6.1)	179 (23.9)	—	—	—	—	—	—	—	—	—
	>3.0%	43 (87.8)	311 (41.5)	617 (77.2)	93.5 (82.1 to 98.6)	45.5 (41.4 to 49.7)	0.89 (0.83 to 0.96)	12.1 (8.9 to 16.0)	98.9 (96.7 to 99.8)	−52.2 (−68.8 to −35.6), P<0.001	50.8 (46.5 to 55.1), P<0.001	617
	<3.0% (secondary)	3 (6.1)	260 (34.7)									
	>10.0%	41 (83.7)	142 (18.9)		89.1 (76.4 to 96.4)	75.1 (71.4 to 78.6)		22.4 (16.6 to 29.1)	98.8 (97.3 to 99.6)	−47.8 (−64.4 to −31.2), P<0.001	21.2 (17.4 to 25.0), P<0.001	617
	<10.0% (primary)	5 (10.2)	429 (57.2)									
IOTA sRRisk model	Missing	2 (4.1)	129 (17.2)	—	—	—	—	—	—	—	—	—
	>3.0%	41 (83.7)	231 (30.8)	668 (83.6)	87.2 (74.3 to 95.2)	62.8 (58.9 to 66.6)	0.86 (0.80 to 0.93)	15.1 (11.0 to 19.9)	98.5 (96.7 to 99.4)	−44.7 (−61.0 to −28.3), P<0.001	33.6 (29.5 to 37.7), P<0.001	666
	<3.0% (secondary)	6 (12.2)	390 (52.0)									
	>10.0%	39 (79.6)	149 (19.9)		83.0 (69.2 to 92.4)	76.0 (72.4 to 79.3)		20.7 (15.2 to 27.2)	98.3 (96.7 to 99.3)	−40.4 (−57.8 to −23.1), P<0.001	20.4 (16.6 to 24.1), P<0.001	666
	<10.0% (primary)	8 (16.3)	472 (62.9)									
IOTA simple rules	Missing	2 (4.1)	124 (16.5)	—	—	—	—	—	—	—	—	—
	Malignant	24 (49.0)	25 (3.3)	553 (69.2)	75.0 (56.6 to 88.5)	95.2 (93.0 to 96.9)	0.85 (0.77 to 0.93)	49.0 (34.4 to 63.7)	98.4 (96.9 to 99.3)	−28.1 (−49.1 to −7.2), P=0.01	2.3 (−0.1 to 4.7), P=0.06	551
	Benign	8 (16.3)	496 (66.1)									
	Inconclusive	15 (30.6)	105 (14.0)	—	—	—	—	—	—	—	—	—
CA 125	Missing	0 (0.0)	4 (0.5)	—	—	—	—	—	—	—	—	—
	>87 IU/mL	27 (55.1)	82 (10.9)	795 (99.5)	55.1 (40.2 to 69.3)	89.0 (86.5 to 91.2)	0.80 (0.72 to 0.87)	24.8 (17.0 to 34.0)	96.8 (95.2 to 98.0)	−10.6 (−21.6 to 0.3), P=0.06	6.9 (4.6 to 9.2), P<0.001	672
	<87 IU/mL	22 (44.9)	664 (88.5)									

OC=ovarian cancer; CI=confidence interval; AUC=area under the curve; CA 125=cancer antigen 125; IOTA=International Ovarian Tumour Analysis; sRRisk=simple rules risk; RMI 1=Risk of Malignancy Index 1; ROMA=Risk of Malignancy Algorithm; ADNEX=Assessment of Different Neoplasias in the Adnexa.

Ovarian cancer present includes primary invasive ovarian malignant neoplasm or a diagnosis of ovarian cancer in the past 12 months; ovarian cancer absent includes a diagnosis of benign, normal, or absent in the past 12 months. 58 (6.8%) women had a diagnostic category of “other” and were not included in the analysis. Women with missing index test data were excluded from both primary and secondary analyses. Participants with missing or inconclusive reference standard results were excluded from the primary outcome definition (presence or absence of ovarian cancer) but were included in the secondary outcome (presence of any cancer) to make the best use of participant data. Differences in sensitivities and specificities are not exact (A-B) for test A and test B, since different numbers of participants are included in each index test analysis, and only those with non-missing index test data for both index tests were included in the calculation of differences.

The pattern of values of sensitivity and specificity across the tests showed a threshold effect, with tests with the lowest sensitivity having the highest specificity, and vice versa (table 3). Risk of Malignancy Index 1 at thresholds of 200 and 250 had the lowest sensitivities of 48.9% (95% CI 34.1% to 63.9%) and 42.6% (95% CI 28.3% to 57.8%), respectively. Compared to Risk of Malignancy Index 1 at 250, CA 125 and all other models had higher sensitivity (CA 125: 55.1%, 95% CI 40.2% to 69.3%, P=0.06; IOTA simple rules: 75.0%, 56.6% to 88.5%, P=0.01; Risk of Malignancy Algorithm at 11.4% threshold: 79.2%, 65.0% to 89.5%, P<0.001; IOTA simple rules risk at 10% threshold: 83.0%, 69.2% to 92.4%, P<0.001; IOTA ADNEX at 10% threshold: 89.1%, 76.4% to 96.4%, P<0.001). Risk of Malignancy Index 1 at thresholds of 200 and 250 had the highest specificities of 95.4% (95% CI 93.4% to 96.9%) and 96.5% (94.7% to 97.8%), respectively. All other tests had lower specificity than Risk of Malignancy Index 1 at 250: IOTA simple rules 95.2%, 93.0% to 96.9%, P=0.06; CA 125 at the 87 IU/mL 89.0%, 86.5% to 91.2%, P<0.001; IOTA simple rules risk at 10% threshold 76.0%, 72.4% to 79.3%, P<0.001; IOTA ADNEX at 10% threshold 75.1%, 71.4% to 78.6%, P<0.001; Risk of Malignancy Algorithm at 11.4% threshold 73.1%, 69.6% to 76.3%, P<0.001).

**Fig 3:**
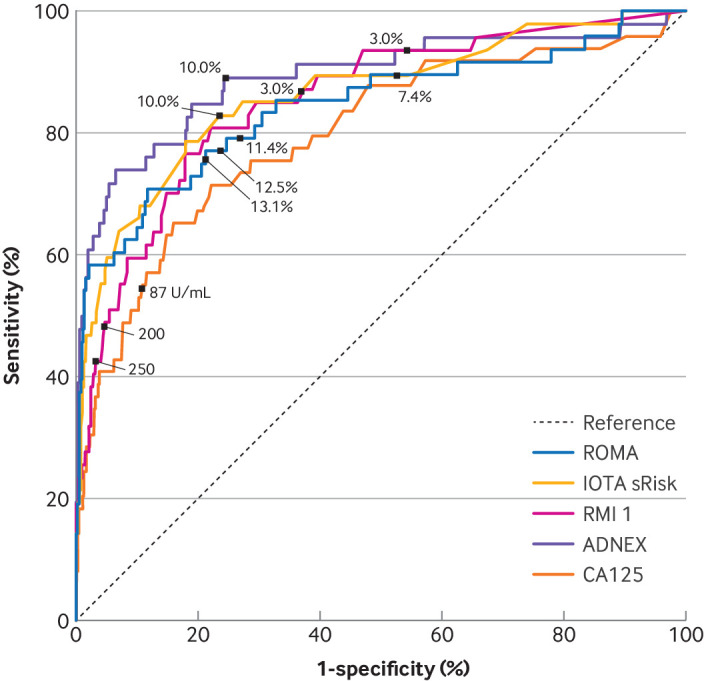
Receiver operating characteristic plot of the index test combinations for the primary outcome in cohort 1 of the ROCkeTS study (to determine accuracy of diagnostic tests for ovarian cancer versus benign or normal in premenopausal women with non-specific symptoms and abnormal test results). Plot produced by considering participants with available data for each index test combination separately. Thus, the number of participants used for each receiver operating characteristic curve varies. IOTA simple rules model was not included in the receiver operating characteristic plot because some participants received inconclusive results. CA 125=Cancer Antigen 125; IOTA=International Ovarian Tumour Analysis; sRRisk=simple rules risk; RMI 1=Risk of Malignancy Index 1; ROMA=Risk of Malignancy Algorithm; ADNEX=Assessment of Different Neoplasias in the Adnexa

**Fig 4:**
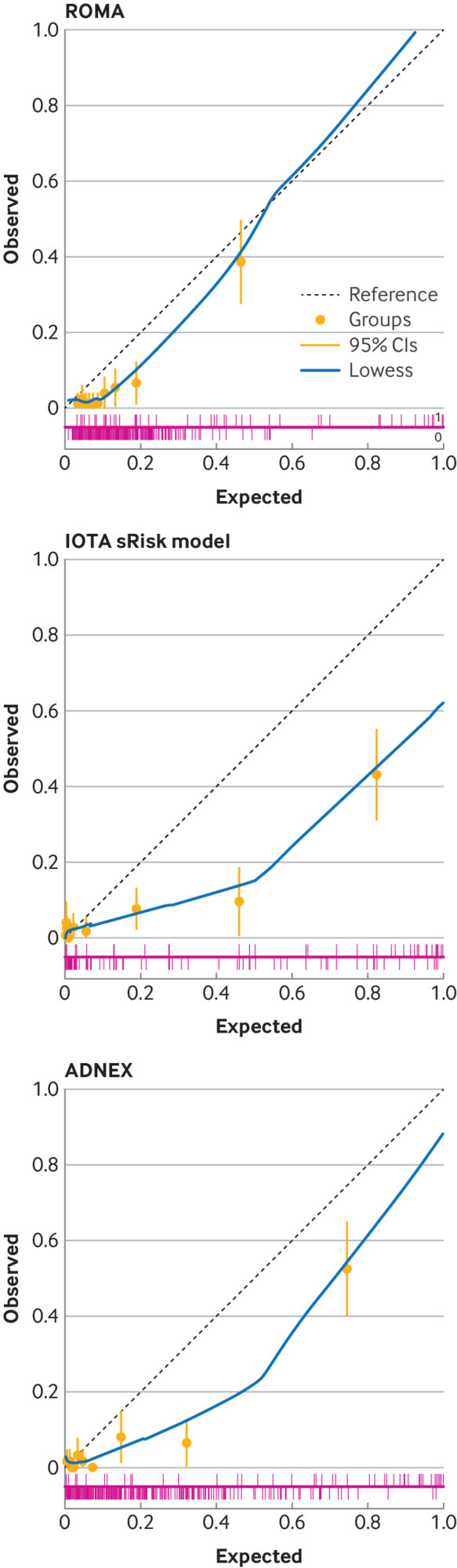
Calibration plots for ROMA, IOTA sRRisk, and ADNEX of the primary outcome in cohort 1 of the ROCkeTS study (to determine accuracy of diagnostic tests for ovarian cancer in premenopausal women with non-specific symptoms and abnormal test results). CI=confidence interval; IOTA=International Ovarian Tumour Analysis; sRRisk=simple rules risk; ROMA=Risk of Malignancy Algorithm; ADNEX=Assessment of Different Neoplasias in the Adnexa. For risk prediction models, the calibration was shown visually by grouping women into deciles ordered by predicted risk and considering the agreement between the mean predicted risk and the observed events in each decile. The value for the calibration slope should ideally be one signifying perfect agreement between the predicted probabilities and the observed probabilities. A calibration slope <1.0 indicates that a model overpredicts, while a calibration slope >1 would underpredict

IOTA ADNEX demonstrated the highest global accuracy with a C index (area under the curve) of 0.89 (95% CI 0.83 to 0.96), followed by IOTA simple rules risk (0.86 (0.80 to 0.93), Risk of Malignancy Index 1 (0.85 (0.79 to 0.91)), IOTA simple rules (0.85 (0.77 to 0.93)), Risk of Malignancy Algorithm (0.84 (0.77 to 0.92)), and CA 125 (0.80 (0.72 to 0.87); table 3, fig 3). Calibration of the models for Risk of Malignancy Algorithm, IOTA ADNEX, and IOTA simple rules risk model demonstrated underprediction (fig 4).

All index tests had high negative predictive values, ranging from 95.7% (95% CI 93.8% to 97.2%) for Risk of Malignancy Index 1 at a threshold of 250 to 98.9% (96.7% to 99.8%) for IOTA ADNEX at a 3% threshold, driven by the low prevalence. Positive predictive values were from 10.4% (7.6% to 13.7%) for Risk of Malignancy Algorithm at 7.4% to 49.0% (34.4% to 63.7%) for IOTA simple rules (table 3).

### Presence of any cancer

In cohort 1, 86 (10.0%) of 857 women received a diagnosis of the secondary outcome definition: the presence of any cancer including borderline ovarian tumours (supplementary appendix 1, supplementary table 2). Estimates of sensitivity were lower than for the primary outcome: sensitivity values were 4% lower for Risk of Malignancy Index 1 (at the 250 threshold) and 14% lower for IOTA ADNEX. Risk of Malignancy Index 1 at 250 threshold reported the lowest sensitivity of 39.0% (95% CI 28.4% to 50.4%), and IOTA ADNEX reported the highest sensitivity of 75.3% (64.5% to 84.2%). Specificity values for the secondary outcome were very similar to those for the primary outcome.

Risk of Malignancy Index 1, Risk of Malignancy Algorithm, IOTA ADNEX, and IOTA simple rules risk reported very similar global accuracy C index values of 0.81, while values were lower for IOTA simple rules (0.76) and CA 125 (0.73) (supplementary appendix 1, supplementary table 2, supplementary fig 1A). The Risk of Malignancy Algorithm model demonstrated good calibration, while the IOTA ADNEX and IOTA simple rules risk probabilities were underpredictions (supplementary appendix 1, supplementary fig 1B).

For the secondary outcome (presence of any cancer), negative predictive values ranged from 91.8% (Risk of Malignancy Index 1 at a threshold of 250) to 97.9% (Risk of Malignancy Algorithm at a threshold of 7.4%), lower than for the primary outcome (diagnostic accuracy at predicting primary invasive ovarian cancer). Positive predictive values ranged from 17.0% (Risk of Malignancy Algorithm at a threshold of 7.4%) to 56.9% (IOTA simple rules).

### Sensitivity analyses

For the primary outcome, the accuracy estimates were comparable in sensitivity analyses that combined participants in the pre-protocol change cohort and post-protocol change cohort into a combined cohort, with only small changes in point estimates but no changes in the performance ranking (supplementary appendix 1, supplementary table 3, supplementary figs 2A and 2B), and in analyses using imputation for missing data (supplementary appendix 1, supplementary table 4).

Sensitivity analyses of the secondary outcome showed consistent results from the combined cohort (supplementary appendix 1, supplementary table 5, supplementary figs 3A and 3B) and in analyses using imputation for missing data (supplementary appendix 1, supplementary table 6).

We also analysed the diagnostic accuracy of the index tests, including borderline tumours with benign tumours as normal in the combined cohort analysis: our findings were consistent with main results (supplementary appendix 1, supplementary table 7). Results according to quality assurance passed sonographers or high volume recruiting centres were also consistent with findings of the main primary and secondary analyses (data not submitted but available on request).

### Post hoc analysis of the Ovarian-Adnexal Reporting and Data System 

In post hoc analyses of the combined cohort, we compared the primary outcome (supplementary appendix 1, supplementary table 8) and secondary outcome (supplementary appendix 1, supplementary table 9) for Ovarian-Adnexal Reporting and Data System at a 10% threshold with Risk of Malignancy Index 1 at 250. The Ovarian-Adnexal Reporting and Data System demonstrated better sensitivity than Risk of Malignancy Index 1 at the 250 threshold, but gave lower specificity for both outcomes. For the primary outcome, the Ovarian-Adnexal Reporting and Data System at 10% threshold had a sensitivity of 81.3% (95% CI 69.5% to 89.9%) and a specificity of 82.2% (79.5% to 84.6%), and a sensitivity of 68.9% (60.3% to 76.7%) and a specificity of 82.2% (79.5% to 84.6%) for the secondary outcome. 

## Discussion

### Statement of principal findings

The ovarian cancer prevalence in premenopausal women observed in our study is low (5.7% in the pre-protocol cohort 1, 7% in the combined cohort). Only 1% of women referred for urgent suspected cancer who are younger than 40 years old receive diagnoses of ovarian cancer.^41^ Achieving a high sensitivity (ie, minimising false negative (missed) diagnoses) is therefore important and challenging. Conversely, reducing unnecessary investigations and surgery owing to false positive diagnoses (ie, maintaining test specificity) is also important. The desire to preserve fertility and ovarian function is also likely to be valuable to these women in this group: 90 women (7.4%) were trying to achieve pregnancy, and only 345 (33.0%) were using contraception.

Our study investigated diagnostic tests for ovarian cancer in premenopausal women with symptoms and abnormal CA 125 levels, ultrasound scan results, or both. The majority of women in this study were referred from primary care through the urgent suspected cancer pathway to hospital clinics. Results showed that the current UK standard triage test, Risk of Malignancy Index 1 at a threshold of 250, has a poor sensitivity of 42.6% (95% CI 28.3% to 57.8%), despite a good specificity of 96.5% (94.7% to 97.8%). IOTA ADNEX at thresholds of 10% and 3%, Risk of Malignancy Algorithm at 11.4% and 7.4%, and IOTA simple rules risk models at 10% and 3% significantly improve on Risk of Malignancy Index 1’s sensitivity but with a significant fall in specificity. Compared to Risk of Malignancy Index 1, IOTA ADNEX at 10% achieved the highest sensitivity at 89.1% (95% CI 76.4% to 96.4%) with a relatively limited loss of specificity, 75.1% (71.4% to 78.6%).

These results were consistent for the detection of primary ovarian cancer, metastatic cancers, and borderline tumours combined (secondary outcome analysis), and across sensitivity analyses.

Among comparator tests, IOTA simple rules appeared to have better than Risk of Malignancy Index 1 to 75.0% (95% CI 56.6% to 88.5%) while maintaining a high specificity of 95.2% (93.0% to 96.9%). However, IOTA simple rules classified 120 of 799 women (15.0%) as having inconclusive results. Inconclusive results are rarely random; they typically represent hard-to-diagnose, borderline presentations with a higher underlying risk of malignancy. Excluding these results introduces spectrum biases and missing-not-at-random biases, making sensitivity and specificity appear better than would be observed in practice.

After the study concluded, we were able to undertake a post hoc analysis of the Ovarian-Adnexal Reporting and Data System at a 10% threshold, which gave a sensitivity of 81.3% (95% CI 69.5% to 89.9%) and a specificity of 82.2% (79.5% to 84.6%), with a smaller gain in sensitivity than IOTA ADNEX but higher specificity. Further investigation of the performance of the Ovarian-Adnexal Reporting and Data System in a prospective study is needed.

### Comparison with existing literature

We searched OVID MEDLINE, OVID EMBASE, and Cochrane Library (to 14 July 2025) using terms: ROMA, IOTA ADNEX, ORADS, IOTA simple rules, and RMI 1 to identify optimal risk prediction models for ovarian cancer in premenopausal women. No head-to-head prospective comparison of all tests was identified, and existing studies were predominantly conducted in high prevalence settings with expert ultrasound operators, limiting generalisability to non-specialist, primary care, or community settings

Our 2022 Cochrane systematic review of risk prediction models for the diagnosis of ovarian cancer demonstrated a pattern of results in premenopausal women similar to our findings in the ROCkeTS study: a higher sensitivity but lower specificity of Risk of Malignancy Algorithm (27 studies, 4463 participants) and IOTA ADNEX (4 studies, 1696 participants) than Risk of Malignancy Index 1 (17 studies, 5233 participants).^22^ Differences in accuracy estimates can be explained by the highly selected participants in included studies (mean ovarian cancer prevalence 16-27%) compared to a prevalence of 5.7% for our main analysis. The results of our study in premenopausal women are also consistent with our previous report from postmenopausal women, wherein we identified IOTA ADNEX ultrasound as the triage test with the highest sensitivity gain over risk of malignancy index triage and a reduction of specificity comparable to other comparator tests.^23^

### Implications for practice for clinicians and policymakers

Our results should be interpreted in the context of current practice. We recruited participants predominantly from urgent suspected cancer pathway referrals (67%), and 60% of primary ovarian cancer diagnoses were stage 1 or 2 (confined to the pelvis), meaning that the cancer is likely curable with standard of care treatment.^42^ A risk prediction model with high sensitivity would improve survival, ensuring women with ovarian cancer are triaged appropriately to receive surgery from trained specialist gynaecological cancer surgeons while enabling women identified as low risk to be managed without surgery and with reassurance alone.^14^

Data from this study demonstrate high surgery rates in women triaged using Risk of Malignancy Index 1 (64.7%, 551/857 patients) despite its relatively high specificity, at least in part because current guidance advocates surgery even in premenopausal women triaged as low risk because of the known low sensitivity of Risk of Malignancy Index 1 in this group. Balancing gains in sensitivity to detect ovarian cancer at an early stage against reductions in specificity, and given the limitations of the IOTA simple rules model, we recommend IOTA ADNEX at 10% to replace Risk of Malignancy Index 1 at the 250 threshold as the UK standard of care ovarian cancer triage test in secondary care. The sensitivity and specificity of the IOTA ADNEX ultrasound model was achieved using non-specialist, appropriately trained, certified, and quality assured sonographers. IOTA ultrasound training resources have been established through our study and are available for all NHS staff.^43^

We have previously reported a high level of anxiety in women referred through urgent suspected cancer pathways, which persists at 12 month follow-up after referral, despite the patient not receiving a cancer diagnosis.^41^ In recommending IOTA ADNEX with high sensitivity and a reduction in specificity, we are aware of one important difference between our trial and how the findings will be implemented in practice. In this study, we evaluated IOTA ADNEX model performance in all patients recruited to robustly evaluate algorithm performance. However, for implementation in practice, we would recommend a two step strategy, initially triaging out women with a benign appearance on scan (<1% risk of cancer over two years) and then using IOTA ADNEX to calculate risk of ovarian cancer in the remaining women. This approach has been shown to have better specificity with an area under the curve of 0.94 (95% CI 0.92 to 0.96), sensitivity of 91.0% (84.5% to 95.0%) and specificity of 85.6% (81.4% to 89.1%).^44^ ^45^ ^46^ For patients with IOTA ADNEX scores of 10-50%, additional magnetic resonance imaging can help prevent unnecessary surgery. We have previously discussed strategies needed to successfully implement IOTA ADNEX ultrasound and a flowchart for practice (supplementary appendix 1, supplementary fig 4).^47^ Our results also suggest that earlier, accurate ovarian cancer diagnosis is possible using IOTA ADNEX ultrasound as a first test in primary care, potentially concurrent with CA 125 testing. This approach needs evaluating in further research (supplementary appendix, supplementary fig 5).

### Strengths and limitations

In our multi-site, blinded, prospective head-to-head comparison study, we looked at all commonly used candidate risk prediction models for the diagnosis of ovarian cancer in newly presenting premenopausal women. We minimised selection bias by recruiting through the UK National Cancer Research Network infrastructure. Quality assured index tests were compared against a common reference standard and a predefined statistical analysis plan included appropriate handling of missing data. Differentiation between primary (ovarian cancer) and secondary (all cancer types including borderline) outcomes allowed investigation of test performance without inflation by inclusion of borderline tumours, which, although common in younger women, have little impact on survival. The study mirrored real life and practice scenarios, wherein some patients are referred to undergo surgery and others are kept under surveillance or discharged. Including a 12 month follow-up allowed us to investigate false negatives of risk prediction models that would be used in clinical care to make triage decisions in women with suspected ovarian cancer.

In contrast to previous studies, the low prevalence of ovarian cancer in our study is applicable to a primary care referred population^22^ and most scans were performed by level 2, non-medical NHS sonographers rather than clinically qualified experts (ie, gynaecologists or radiologists) participating from centres of excellence.^48^

Although our recruitment is consistent with UK census patterns (ie, comprising mainly white patients) results may be less applicable to people of different ethnicities. Although we are unable to identify differences in patient characteristics between the pre-protocol change cohort and post-protocol change cohort, our decision to restrict recruitment to presurgical patients (following observation of low ovarian cancer prevalence in this group in an interim analysis) is likely to have led to systematic differences between the two cohorts. Further, not scheduling an additional hospital visit for a transvaginal IOTA ultrasound in women in the post-protocol change cohort—to reduce risk during the covid-19 pandemic and to improve recruitment—led to high data missingness, particularly of ultrasound variables data.

Although we encouraged sites to perform the IOTA ultrasound scan at the same time as routine scans for clinical care, we did not collect information on how this was delivered across sites. It is possible that the sonographers who completed the scan to calculate Risk of Malignancy Index 1 were different in some sites from the sonographers who collected the IOTA ultrasound scan data.

Despite these issues, the consistency of results across the pre-protocol cohort, combined cohort, and imputed analyses across primary and secondary analyses demonstrates the robustness of our study's findings. The challenges of conducting this study and estimating sample size are common in evaluations of test accuracy in low prevalence populations. We believe that our study has learnings for other researchers designing a diagnostic test accuracy study to diagnose rare events in diseases with commonly occurring symptoms.

### Implications for research

The performance of risk prediction models in ethnically diverse populations needs further research. Alternative methods of evaluating the impact of diagnostic test use on clinical utility or net benefit may be highly relevant and will be conducted as next steps in the ROCkeTS study.^49^ Exploring the effect of threshold on test performance using the area under the curve may have a role in determining optimal trade-offs in sensitivity and specificity. However, limitations of this approach include not providing the threshold needed to realise the optimal trade-off. This is essential for clinicians and health systems to make decisions about further management. The use of decision curve analysis to compare decisions at the same thresholds across risk prediction models was not possible because Risk of Malignancy Index 1 does not produce probabilities.

Long waiting times for ultrasound scans the absence of standardisation, and quality assurance are key challenges to achieving IOTA ultrasound scan implementation at scale in the NHS.^8^ Artificial intelligence enabled solutions for ultrasound scans, plus quality assurance and training for sonographers, may deliver timely ultrasound scan availability in practice for women with non-specific symptoms and substantially improve outcomes by early diagnosis. The impact of artificial intelligence enabled ultrasound scans with IOTA ADNEX in primary care practice on expediting diagnostic intervals and improving early detection of ovarian cancer needs investigation. Understanding facilitators and barriers to implementing change in diagnostic pathways, including a one stop model for IOTA ultrasound scans, is currently being investigated in the SONATA study (NCT06129968).

In a post hoc analysis, when Ovarian-Adnexal Reporting and Data System and IOTA ADNEX were compared independently to Risk of Malignancy Index 1 at the 250 threshold, the Ovarian-Adnexal Reporting and Data System at the 10% threshold achieved a smaller gain in sensitivity but a lower drop in specificity than IOTA ADNEX at the 10% threshold. However, the latest version of Ovarian-Adnexal Reporting and Data System (version 2) with additional variables was not evaluated.^31^ Prospective multicentre research studies investigating the diagnostic accuracy of the Ovarian-Adnexal Reporting and Data System for ovarian cancer are necessary.

### Conclusion

The current triage test for ovarian cancer, Risk of Malignancy Index 1, demonstrates poor sensitivity in premenopausal women and should be replaced. IOTA ADNEX at the 10% threshold, delivered by trained and quality assured NHS sonographers, achieves significantly higher sensitivity with limited specificity reduction in a real world cohort and should be considered the new standard of care for secondary care triage. Primary care implementation could potentially improve survival through earlier detection in symptomatic premenopausal women (supplementary appendix 1, supplementary fig 5), but this requires further research alongside investment in sonographer training and quality assurance.

**What is already known on this topic**

Diagnosing ovarian cancer in premenopausal women is challenging: ovarian cancer is rare, while symptoms (elevated CA 125 serum levels and physiological ovarian cysts on ultrasound) are common
Current standard of care risk prediction model used to triage women with ovarian cysts into low or high risk of ovarian cancer is the Risk of Malignancy Index 1
We searched OVID MEDLINE, OVID EMBASE, and Cochrane Library (to 14 July 2025) using terms: ROMA, IOTA ADNEX, ORADS, IOTA simple rules, and RMI 1 to identify optimal risk prediction models for ovarian cancer in premenopausal women
No head-to-head prospective comparison of all tests was identified, and existing studies were predominantly conducted in high prevalence settings with expert ultrasound operators, limiting generalisability to non-specialist, primary care, or community settings

**What this study adds**

We conducted a prospective head-to-head test accuracy study of common risk prediction models in premenopausal women presenting to secondary care with symptoms, abnormal CA 125, and abnormal ultrasound
A study representative of real world populations with lower ovarian cancer prevalence (5.7%). Ultrasound scans were performed primarily by NHS sonographers, enhancing applicability to practice
Risk of Malignancy Index 1 at the 250 threshold shows poor sensitivity (42.6%) but high specificity (96.5%) in premenopausal women, and alternative tests improved sensitivity at the expense of reduced specificity compared to Risk of Malignancy Index 1 at the 250 threshold
IOTA ADNEX at 10% threshold demonstrates the highest sensitivity (89%) with specificity (75%) compared to other evaluated tests and is recommended for practice

## Data Availability

The dataset generated including deidentified patient data and samples analysed during the study, along with additional material such as protocol, statistical analysis plan is available at Birmingham Clinical Trials Unit, University of Birmingham after date of publication. The dataset is not publicly available but maybe obtained on request to SS, review by Project oversight group, NIHR, ethics approval, and after fulfilling all data transfer requirements.

## Web extra

Extra material supplied by authors

Supplementary appendix 1: Supplementary materials

Supplementary appendix 2: ROCkeTS collaborator group

## Notes

Contributors: SS, CD, SM, and JD conceptualised and designed the study. SS, SJ, PS, and RS-V recruited to the study and collaborators CR, RO, and LS coordinated the study. SM, JD, KS, and RA analysed results from the study. DT and TB conducted ultrasound quality assurance and training. BVC provided insight into analysis of ultrasound models. SK, RN, UM, and AG-M provided input into study design and conduct. HS provided patient perspectives throughout the study from the grant application, study conduct, and interpretation of results. All authors reviewed the results and the manuscript. JD, RA, KS, LS, and RO directly accessed and verified the underlying data reported in the manuscript. All authors confirm that they had full access to all the data in the study and accept responsibility to submit for publication. The corresponding author wrote the manuscript and used artificial intelligence large language models to edit the manuscript. SS is guarantor of paper. The guarantor accepts full responsibility for the work and the conduct of the study, had access to the data, and controlled the decision to publish. The corresponding author attests that all listed authors meet authorship criteria and that no others meeting the criteria have been omitted. A list of collaborators in the ROCkeTS group can be found in supplementary appendix 2.

Transparency: The lead author (the manuscript's guarantor) affirms that the manuscript is an honest, accurate, and transparent account of the study being reported; that no important aspects of the study have been omitted; and that any discrepancies from the study as originally planned (and, if relevant, registered) have been explained.

Dissemination to participants and related patient and public communities: These results have been presented at the International Gynaecological Cancer Society meeting in Dublin, October 2024, at the Royal College of Obstetricians and Gynaecologists annual meeting in Oman October 2024 and at multiple national meetings. Results will be summarised in lay language and placed on the ROCkeTS trial website. We do not plan to contact participants individually to communicate the results of the study

Provenance and peer review: Not commissioned; externally peer reviewed.
