# Supplementary material for: Diagnostic tests for ovarian cancer in premenopausal women with non-specific symptoms (ROCkeTS): prospective, multicentre, cohort study
Source: BMJ. 2026 Jan 29;392:e083912. doi: 10.1136/bmj-2024-083912 (PMC12849051; doi:10.1136/bmj-2024-083912)
Supplement: Supplementary file 2 — Supplementary appendix 2: ROCkeTS collaborator group [file suns083912.ww2.pdf]

ROCkeTS collaborator group

Ahmed Abdelbar (Guy's Hospital, Great Maze Pond, London SE1 9RT), Shahram Abdi (Royal Hallamshire Hospital, Glossop Rd, Broomhall, Sheffield S10 2JF), Parveen Abedin (Birmingham Womens Hospital, Mindelsohn Way, Birmingham B15 2TG), Hafez Alawad (Northampton General Hospital, Cliftonville, Northampton NN1 5BD), Victoria Ames (Norfolk & Norwich University Hospital, Colney Lane Norwich NR4 7UY), Moji Balogun (Birmingham Womens Hospital, Mindelsohn Way, Birmingham B15 2TG), Sarah Baron (Royal Sussex County Hospital, Eastern Rd, Brighton and Hove, Brighton BN2 5BE), Tracey Butcher (Royal Preston/Sharoe Green Hospitals, Sharoe Green Ln, Fulwood, Preston PR2 9HT), Sarah Coleridge (Nottingham City Hospital, Hucknall Road, Nottingham, NG5 1PB), Tim Duncan (Norfolk & Norwich University Hospital, Colney Lane Norwich NR4 7UY), Kendra Exley (The James Cook University Hospital, Marton Rd, Middlesbrough TS4 3BW), Ketankumar Gajjar (Nottingham City Hospital, Hucknall Road, Nottingham, NG5 1PB), Fateh Ghazal (Walsall Manor Hospital, Moat Rd, Walsall WS2 9PS), Chellappah Gnanachandran (Northampton General Hospital, Cliftonville, Northampton NN1 5BD), Marianne Hamer (Liverpool Womens Hospital, Crown St, Liverpool L8 7SS), Neil Hebblethwaite (The James Cook University Hospital, Marton Rd, Middlesbrough TS4 3BW), Tracey Hughes (Queen Elizabeth Hospital, Queen Elizabeth Ave, Gateshead NE9 6SX), Karen Jermy (East Surrey Hospital, Canada Ave, Redhill RH1 5RH), Susanne Johnson (Princess Anne Hospital, Coxford Rd, Southampton SO16 5YA), Sonali Kaushik (Royal Sussex County Hospital, Eastern Rd, Brighton and Hove, Brighton BN2 5BE), Patrick Keating (Royal Preston/Sharoe Green Hospitals, Sharoe Green Ln, Fulwood, Preston PR2 9HT), Robert Kent (University Hospital Of North Durham, North Rd, Durham DH1 5TW), Humaira Khan (Birmingham City Hospital, Dudley Rd, Birmingham B18 7QH), Robert Macdonald (Liverpool Womens Hospital, Crown St, Liverpool L8 7SS), Ciara Mackenzie (Hinchingbrooke Hospital, Parkway Hinchingbrooke, Huntingdon PE29 6NT), Julia Maddison (The Royal Victoria Infirmary, Queen Victoria Rd, Newcastle upon Tyne NE1 4LP), Tarang Majmudar (Hinchingbrooke Hospital, Parkway Hinchingbrooke, Huntingdon PE29 6NT), Vivek Malhotra (Watford General Hospital, Vicarage Rd, Watford WD18 0HB), Ranjit Manchanda (The Royal London Hospital, Whitechapel Rd, London E1 1FR), Roger Moshy (Peterborough City Hospital, Edith Cavell Campus, Bretton Gate, Peterborough PE3 9GZ), Hans Nagar (Belfast City Hospital, Lisburn Rd, Belfast BT9 7AB), Adam Naskretski (Nottingham City Hospital, Hucknall Road, Nottingham, NG5 1PB), Julia Palmer (Sheffield Teaching Hospitals, Royal Hallamshire Hospital, Broomhill, Glossop Rd, Sheffield S10 2JF),

Robert Parker (Birmingham City Hospital, Dudley Rd, Birmingham B18 7QH), Selvi Radhikavikram (Watford General Hospital, Vicarage Rd, Watford WD18 0HB), Harinder Rai (Walsall Manor Hospital, Moat Rd, Walsall WS2 9PS), Bruce Ramsay (Peterborough City Hospital, Edith Cavell Campus, Bretton Gate, Peterborough PE3 9GZ), Natalia Rosello (Princess Anne Hospital, Coxford Rd, Southampton SO16 5YA), Michelle Russell (The Royal Victoria Infirmary, Queen Victoria Rd, Newcastle upon Tyne NE1 4LP), Ahmad Sayasneh (Guy's Hospital, Great Maze Pond, London SE1 9RT), Partha Sengupta (University Hospital Of North Durham, North Rd, Durham DH1 5TW), Aarti Sharma (University Hospital Of Wales, Heath Park Way, Cardiff CF14 4XW), Anju Sinha (University Hospital Of Wales, Heath Park Way, Cardiff CF14 4XW), Lavanya Vitta (Royal Sussex County Hospital, Eastern Rd, Brighton and Hove, Brighton BN2 5BE), Mark Willett (Royal Blackburn Teaching Hospital, Haslingden Rd, Blackburn BB2 3HH), Nicholas Wood (Royal Preston/Sharoe Green Hospitals, Sharoe Green Ln, Fulwood, Preston PR2 9HT), Ahmed Darwish (Northampton General Hospital, Cliftonville, Northampton NN1 5BD)
